# Robot-assisted total hip arthroplasty following gene therapy for hemophilia a arthropathy: a case report

**DOI:** 10.1186/s42836-025-00348-5

**Published:** 2025-12-03

**Authors:** Chuanlong Wu, Jing Dai, Zhijie Chen, Yanyan Shao, Xuefeng Wang, Chuan He

**Affiliations:** 1 Department of Orthopaedics, Shanghai Key Laboratory for Prevention and Treatment of Bone and Joint Diseases, Shanghai Institute of Traumatology and Orthopaedics, Ruijin Hospital, Shanghai Jiao Tong University School of Medicine, Shanghai, 200025 China; 2 Department of Laboratory Medicine, Ruijin Hospital, Shanghai Jiao Tong University School of Medicine, Shanghai, 200025 China

**Keywords:** Robot-assisted, Total hip arthroplasty, Hemophilia A, Arthropathy, Gene therapy

## Abstract

**Background:**

Hemophilia A (HA) arthropathy poses a significant clinical challenge, particularly in achieving perioperative hemostasis and surgical precision. Accounting for the majority of hemophilia cases, HA frequently leads to severe secondary joint damage. Although gene therapy holds promise, its clinical application in HA remains limited by challenges to durable efficacy. Crucially, the transient efficacy and unpredictable pharmacokinetics of gene therapy have not been evaluated in major orthopedic surgeries with a high potential for bleeding, such as total hip arthroplasty (THA).

**Case presentation:**

To our knowledge, this is the first documented case of robot-assisted (RA)-THA performed after gene therapy for severe HA-associated hip arthropathy, including a detailed surgical protocol and a postoperative follow-up of over six months. The patient received hemophilia gene therapy 56 weeks prior to surgery. Crucially, no exogenous FVIII supplementation was required preoperatively or intraoperatively. A minimal amount of FVIII was administered postoperatively. No abnormal bleeding events occurred throughout the perioperative period. The patient maintained normal coagulation status, achieved excellent wound healing, and demonstrated excellent functional recovery.

**Conclusion:**

This pioneering case demonstrates the potential safety and efficacy of combining gene therapy with minimally invasive RA-THA for HA hip arthropathy. The integrated approach significantly reduced dependence on exogenous FVIII and enabled precise anatomical reconstruction.

**Supplementary Information:**

The online version contains supplementary material available at 10.1186/s42836-025-00348-5.

## Background

End-stage hemophilic arthropathy continues to pose a formidable clinical challenge, demanding exceptional precision in both hemostatic management and surgical execution [1–3]. Hemophilia A (HA) accounts for 85% of all hemophilia cases [4]. Although gene therapy has demonstrated considerable progress in recent years, its clinical application in HA remains constrained by challenges related to long-term stability and durable efficacy [5–7]. Notably, patients undergoing gene therapy have yet to undergo rigorous testing of transient efficacy and unpredictable pharmacokinetics in major orthopedic surgery with potential high-risk bleeding disorders. Conventional total hip arthroplasty (THA) remains one of the most potentially hemorrhagic orthopedic procedures [8, 9], especially hidden blood loss [10, 11]. This study pioneers a precision treatment protocol for Hemophilia A-associated hip arthropathy: Robot-assisted (RA) minimally invasive THA following gene therapy. To our knowledge, this is the first documented case worldwide of this combined approach, including a detailed surgical protocol and a postoperative follow-up of over six months.

## Case presentation

### Patients information

A 27-year-old male with severe HA enrolled in a gene therapy clinical trial (GS1191-0445) [12], receiving AAV8-mediated hepatic FVIII gene transfer. Following treatment, the FVIII activity level, as measured by the one-stage clotting assay, peaked at 202.7 IU/dL and subsequently declined to a plateau of approximately 60 IU/dL by week 56 [13]. Although gene therapy was successful, at 56 weeks post-treatment, the patient required THA due to end-stage hemophilic arthropathy, causing debilitating pain and restricted mobility.

**Fig. 1:**
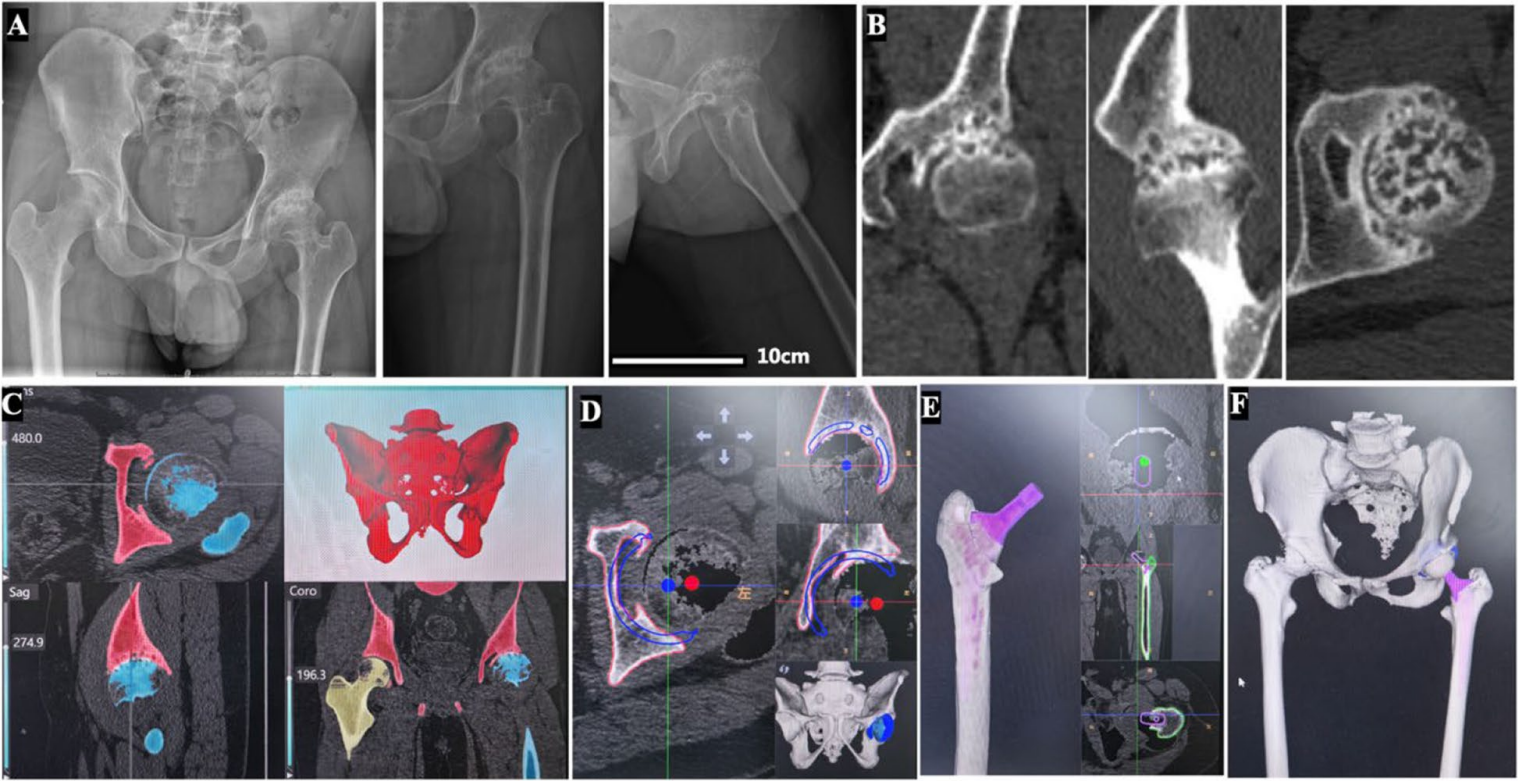
Preoperative imaging and robotic planning. **A**. Preoperative X-ray radiographs: anteroposterior pelvic view (left), anteroposterior left hip view (middle), and lateral left hip view (right) X-rays. The left hip joint shows collapse and deformation of the femoral head with a narrowed joint space and acetabular wear. **B**. Preoperative CT scans: Coronal (left), sagittal (middle), and axial (right) planes. Reveals left femoral head and acetabular deformity with cystic changes. **C**. Preoperative robotic system-assisted (RA) CT segmentation. **D**. Preoperative RA prosthesis planning of the acetabular component based on CT scans. **E**. Preoperative RA prosthesis planning of the femoral component based on CT scans. **F**. Preoperative RA prosthesis planning via 3D imaging

Preoperative radiographs demonstrated severe cystic changes and deformity of the left femoral head, accompanied by cystic changes in the acetabulum (Fig. 1A&B). Preoperative planning included CT-based segmentation and modeling using a surgical robotic system (KUNWU®, YUANHUA Robotics, Perception and AI Technologies, Shenzhen, China) (Fig. 1C), followed by prosthetic templating (Fig. 1D–F).

**Fig. 2:**
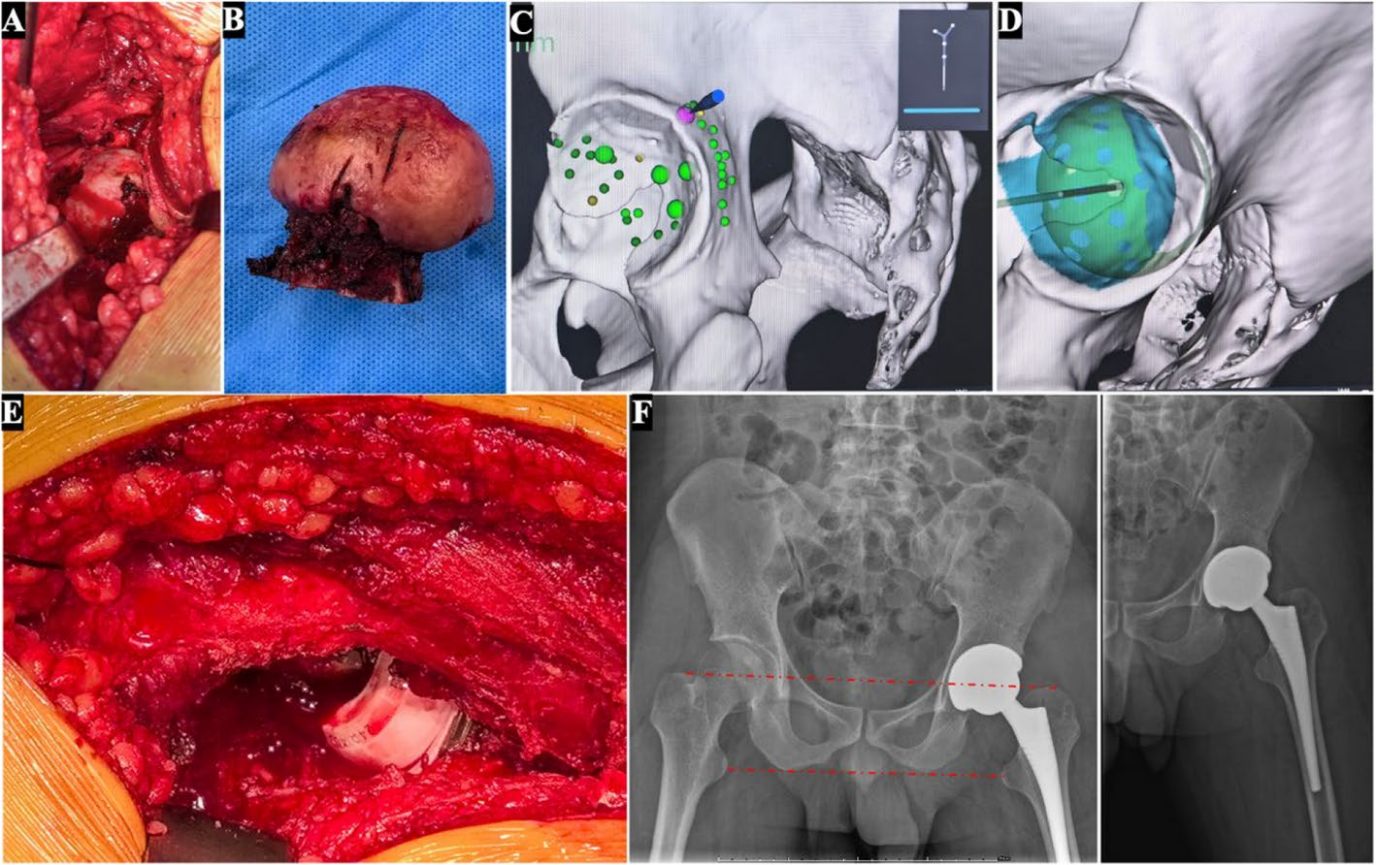
Robotic-assisted (RA) total hip arthroplasty (THA): Intraoperative procedures and surgical field. **A**. Intraoperative view of the acetabulum. **B**. Resected femoral head. **C**. Intraoperative registration using the robotic system. **D**. Intraoperative RA acetabular reaming. **E**. Intraoperative view of post-prosthesis placement. **F**. Postoperative anteroposterior pelvic view (left) and anteroposterior left hip view (right) after left THA

A piriformis-sparing posterolateral minimally invasive approach was employed. No abnormal intraoperative bleeding was observed in the surgical field (Fig. 2A&B). The procedure was performed sequentially with intraoperative registration (Fig. 2C), followed by navigated reaming under robotic guidance (Fig. 2D), and prosthesis implantation accordingly (Fig. 2E). Surgical interventions included complete synovectomy of hyperplastic synovium and anatomical reconstruction of the posterior capsuloligamentous structures using non-absorbable sutures. Postoperative imaging in 24 h demonstrated successful restoration of near-equal limb length (discrepancy < 3 mm), with acetabular component positioning at 38° inclination and 18° anteversion—parameters within 2° of ideal targets (Fig. 2F).

Preoperative FVIII levels remained stable at approximately ~ 60 IU/dL. No factor VIII supplementation was administered pre- or intraoperatively. The perioperative blood loss was approximately 300 mL. On postoperative day 1, a drainage volume of 150 mL combined with a drop in hemoglobin (from 147 g/L preoperatively to 108 g/L) prompted the transfusion of 200 mL of fresh frozen plasma and 2 units of red blood cells. The drain output then dropped to 10 mL on day 2 and was therefore removed. Postoperatively, due to a decline in FVIII:C levels and a progressive drop in hemoglobin, 1000-IU bolus doses of recombinant FVIII were administered on days 1 and 2. This regimen successfully raised the FVIII:C level to 106.9 IU/dL by day 3, which maintained levels above 75 IU/dL through day 14 and above 50 IU/dL until 6 months, with no coagulation abnormalities observed [13]. Thromboprophylaxis was commenced on postoperative day 5, following the confirmation of sustained FVIII recovery. The patient received low-molecular-weight heparin (4000 IU daily) for 3 days, followed by rivaroxaban (10 mg once daily) upon discharge for a further 10 days [13].

**Fig. 3:**
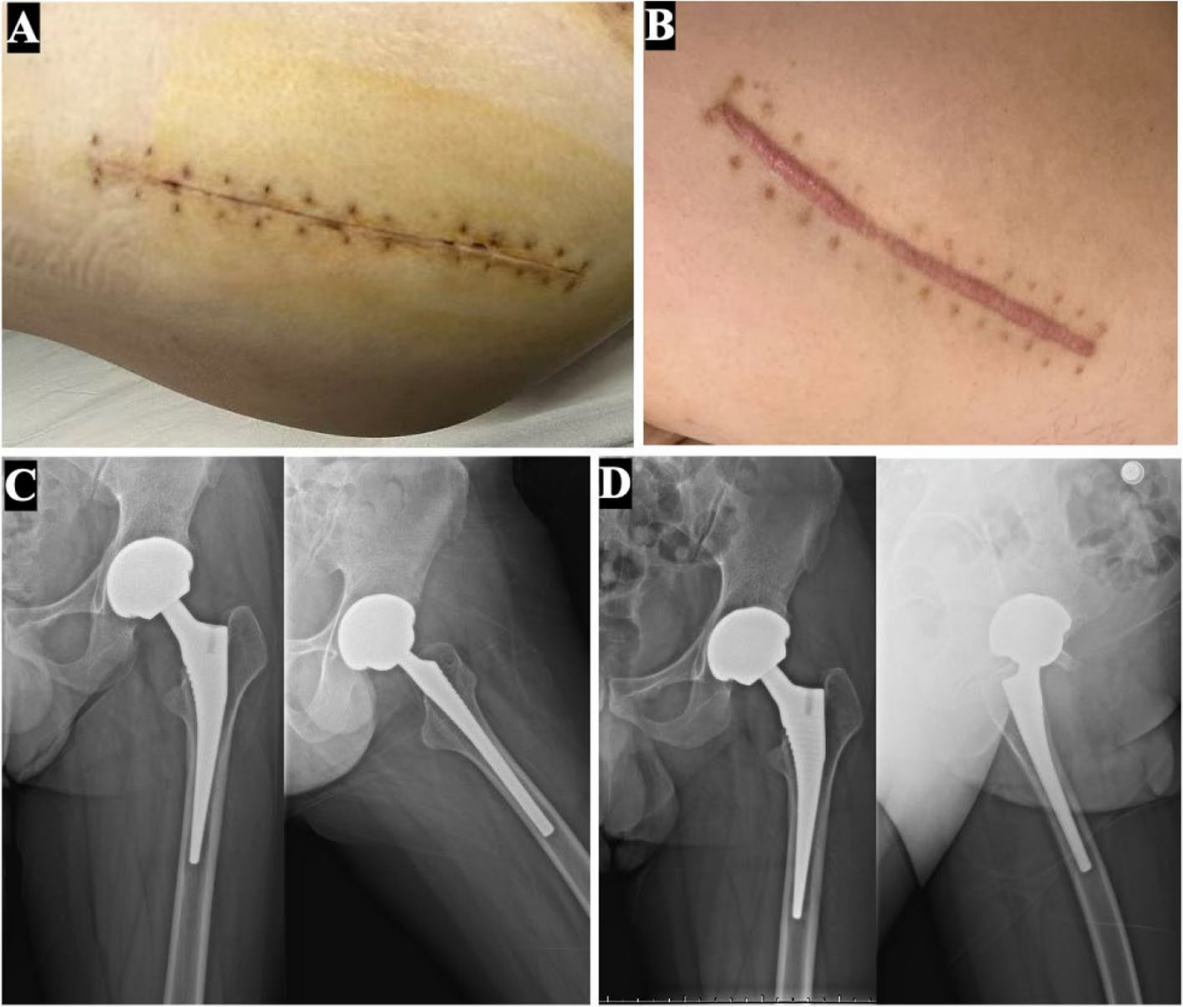
Postoperative evaluation and follow-up. **A**. Wound condition at 2 weeks postoperatively, showing good healing. **B**. Wound condition at 6 months postoperatively, indicating excellent healing. **C**. Six-week postoperative follow-up: anteroposterior left hip view (left) and lateral left hip view (right) after left THA, showing stable prosthesis positioning. **D**. Six-month postoperative follow-up: anteroposterior left hip view (left) and lateral left hip view (right) after left THA, confirming optimal prosthesis positioning

With the assistance of a physical therapist, the patient was able to sit up at the bedside, stand, and attempt walking with a walker on postoperative day 1. On postoperative day 2, the patient proceeded to perform normal walking exercises with a walker under the guidance of the rehabilitation therapist. Specifically, the patient exhibited a transient elevation in liver function tests on postoperative day 5. Glucocorticoids were administered on an as-needed basis in response to increased ALT levels, and liver function returned to normal within two weeks. Throughout this period, the patient denied any discomfort, and no other abnormal complications were observed. Serial wound assessments demonstrated primary intention healing without erythema/drainage at 2-week and 6-month follow-ups (Fig. 3A&B). Radiographic surveillance at 6 weeks and 6 months postoperatively showed stable osseointegration with no evidence of migration (radiolucent line < 1 mm in all zones) (Fig. 3C&D). Functional outcomes at 6 months were favorable, as evidenced by the Harris Hip Score increasing from 62.2 preoperatively to 88.4 postoperatively, and the WOMAC score rising from 65.63 preoperatively to 91.67 postoperatively, though gait abnormalities persisted due to hemophilic arthropathy in the ipsilateral ankle (Supplementary Video 1–2 and Fig. S1).

## Discussion and conclusions

Advanced hemophilic arthropathy presents a significant challenge for orthopedic surgeons, both in terms of perioperative coagulation management and surgical difficulty [1–3]. To our knowledge, this case report may represent a novel precision treatment strategy that integrates gene therapy with RA-THA. The perioperative course suggests potential safety and feasibility, with a dramatic 96% reduction in exogenous FVIII requirements [13].

Recent years have witnessed significant success in gene therapy for hemophilia, particularly for Hemophilia B, where treatment has demonstrated better durability and stability [14–17]. Xue et al. [18] reported a case of a Hemophilia B patient who underwent total knee arthroplasty following gene therapy. Notably, the patient required no preoperative clotting factor supplementation; the surgery proceeded smoothly, and the patient maintained a favorable prognosis at 6-week follow-up. However, the prevalence of Hemophilia A is five times higher than that of Hemophilia B, and the efficacy of gene therapy appears less favorable for Hemophilia A. Furthermore, the perioperative blood loss and bleeding risk associated with hip surgery are substantially greater than those for knee surgery [8]. Consequently, the reporting of this case holds immense clinical significance.

This case also employed multiple modalities, including robot-assisted surgery, to enhance surgical precision, minimize perioperative bleeding, and promote rapid recovery. Specifically, the key technical aspects included: 1. Utilizing robotic technology for precise joint reconstruction [19, 20]; 2. Employing a posterolateral minimally invasive approach that preserves the piriformis muscle and ensures complete reconstruction of the posterior joint capsule [21, 22]; 3. Performing a thorough intraoperative synovectomy of diseased tissue. The implementation of these measures facilitated the patient’s rapid recovery.

This study has several limitations that should be considered. First, its nature as a single-case report precludes definitive conclusions regarding the broader efficacy and safety of this combined approach. Second, the follow-up period of approximately six months is insufficient to evaluate the long-term outcomes of both the gene therapy and the arthroplasty. Finally, the absence of a control group makes it impossible to directly compare this strategy against conventional management. Therefore, our findings necessitate further validation through larger, prospective studies or randomized controlled trials.

This case confirms the potential of combining gene therapy and RA-THA to manage advanced hemophilic arthropathy, effectively minimizing the need for exogenous clotting factor. This strategy represents a proof-of-concept that warrants further investigation in larger cohorts to establish its long-term efficacy. We anticipate that broader adoption of gene therapy may enable early intervention to prevent joint deterioration in hemophilia patients, ultimately reducing the need for arthroplasty.

## Supplementary Information

Supplementary Material 1. Video 1: Demonstration of gait at the 6-month postoperative follow-up.

Supplementary Material 2. Video 2: Demonstration of functional recovery, including the ability to don socks, at the 6-month follow-up.

Supplementary Material 3. Fig. S1. Anteroposterior (left) and lateral (right) X-rays of the left ankle joint, revealing hemophilic arthropathy with narrowed joint space.

## Data Availability

All data generated or analyzed during this case study, including clinical details and imaging, are included in this published article. Additional raw data are available from the corresponding author upon reasonable request.

## Abbreviations

HA: Hemophilia A
THA: Total hip arthroplasty
RA: Robot-assisted

## References

[1] Rodriguez-Merchan EC. Aspects of current management: orthopaedic surgery in haemophilia. Haemophilia. 2012;18(1):8–16. 21535324 10.1111/j.1365-2516.2011.02544.x

[2] Fenelon C, Murphy EP, Fahey EJ, Murphy RP, O’Connell NM, Queally JM: Total Knee Arthroplasty in Hemophilia: Survivorship and Outcomes-A Systematic Review and Meta-Analysis. J Arthroplasty 2022, 37(3):581–592 e581. 10.1016/j.arth.2021.10.015 34756977

[3] Wang SH, Chung CH, Chen YC, Cooper AM, Chien WC, Pan RY: Does Hemophilia Increase Risk of Adverse Outcomes Following Total Hip and Knee Arthroplasty? A Propensity Score-Matched Analysis of a Nationwide, Population-Based Study. J Arthroplasty 2019, 34(10):2329–2336 e2321. 10.1016/j.arth.2019.05.062 31337553

[4] Bolton-Maggs PH, Pasi KJ. Haemophilias A and B. Lancet. 2003;361(9371):1801–9. 12781551 10.1016/S0140-6736(03)13405-8

[5] George LA. Hemophilia A gene therapy - some answers, more questions. N Engl J Med. 2023;388(8):761–3. 36812440 10.1056/NEJMe2212347

[6] Ozelo MC, Mahlangu J, Pasi KJ, Giermasz A, Leavitt AD, Laffan M, et al. Valoctocogene roxaparvovec gene therapy for hemophilia A. N Engl J Med. 2022;386(11):1013–25. 35294811 10.1056/NEJMoa2113708

[7] Leebeek FWG, Miesbach W. Gene therapy for hemophilia: a review on clinical benefit, limitations, and remaining issues. Blood. 2021;138(11):923–31. 34232980 10.1182/blood.2019003777

[8] Callaghan JJ, O’Rourke MR, Liu SS. Blood management: issues and options. J Arthroplasty. 2005;20(4 Suppl 2):51–4. 15991130 10.1016/j.arth.2005.03.018

[9] Sehat KR, Evans RL, Newman JH. Hidden blood loss following hip and knee arthroplasty. Correct management of blood loss should take hidden loss into account. J Bone Joint Surg Br. 2004;86(4):561–5. 15174554

[10] Liu X, Zhang X, Chen Y, Wang Q, Jiang Y, Zeng B: Hidden blood loss after total hip arthroplasty. J Arthroplasty 2011, 26(7):1100–1105 e1101. 10.1016/j.arth.2010.11.013 21256705

[11] Cai L, Chen L, Zhao C, Wang Q, Kang P. Influencing factors of hidden blood loss after primary total hip arthroplasty through the posterior approach: a retrospective study. BMC Musculoskelet Disord. 2023;24(1):582. 37461071 10.1186/s12891-023-06716-z PMC10351163

[12] Liu W, Pei X, Xu B, Dai X, Xue F, Chen L, et al. Prophylactic administration of glucocorticoids and tacrolimus in AAV8-F8 gene therapy of severe haemophilia-a achieved a significant long-term efficacy. Blood. 2024;144:3583.

[13] Shao Y, Wu C, Liu W, Liu Y, Ding Q, Wu W, Wang X, Zhang L, He C, Dai J: Perioperative management of total hip arthroplasty in hemophilia A following gene therapy. Haematologica 2025.10.3324/haematol.2025.28807440637777

[14] Pipe SW, Leebeek FWG, Recht M, Key NS, Castaman G, Miesbach W, et al. Gene therapy with etranacogene dezaparvovec for hemophilia B. N Engl J Med. 2023;388(8):706–18. 36812434 10.1056/NEJMoa2211644

[15] Cuker A, Kavakli K, Frenzel L, Wang JD, Astermark J, Cerqueira MH, et al. Gene therapy with fidanacogene elaparvovec in adults with hemophilia B. N Engl J Med. 2024;391(12):1108–18. 39321362 10.1056/NEJMoa2302982

[16] George LA, Monahan PE, Eyster ME, Sullivan SK, Ragni MV, Croteau SE, et al. Multiyear factor VIII expression after AAV gene transfer for hemophilia A. N Engl J Med. 2021;385(21):1961–73. 34788507 10.1056/NEJMoa2104205 PMC8672712

[17] Srivastava A, Abraham A, Aboobacker F, Singh G, Geevar T, Kulkarni U, et al. Lentiviral gene therapy with CD34+ hematopoietic cells for hemophilia A. N Engl J Med. 2025;392(5):450–7. 39655790 10.1056/NEJMoa2410597 PMC11875532

[18] Xue F, Wang P, Yuan Z, Shi C, Fang Y, Liu W, et al. Total Knee Arthroplasty after Gene Therapy for Hemophilia B. N Engl J Med. 2022;387(17):1622–4. 36306204 10.1056/NEJMc2211173 PMC9933182

[19] Zhang Y, Li S, Liu X, Wang W, Zhou Z, Chai W, Qian W, Liu P, Liao H: Robot-Assisted Total Hip Arthroplasty Versus Conventional Surgery in Terms of Surgical Accuracy, Function, Trauma, and Complications: A Prospective, Multicenter, Parallel-Group, Open-Label Randomized Controlled Trial. J Arthroplasty 2025. 10.1016/j.arth.2025.07.029 40701283

[20] Nakamura N, Sugano N, Nishii T, Kakimoto A, Miki H. A comparison between robotic-assisted and manual implantation of cementless total hip arthroplasty. Clin Orthop Relat Res. 2010;468(4):1072–81. 19890680 10.1007/s11999-009-1158-2 PMC2835605

[21] Apinyankul R, Satravaha Y, Mokmongkolkul K, Phruetthiphat OA. Comparison of dislocation and outcome between piriformis-sparing and conventional posterior approach after bipolar hemiarthroplasty for femoral neck fracture in patients over 60 years. J Arthroplasty. 2023;38(4):732–6. 36273711 10.1016/j.arth.2022.10.025

[22] Tan BKL, Khan RJK, Haebich SJ, Maor D, Blake EL, Breidahl WH. Piriformis-sparing minimally invasive versus the standard posterior approach for total hip arthroplasty: a 10-year follow-up of a randomized control trial. J Arthroplasty. 2019;34(2):319–26. 30442467 10.1016/j.arth.2018.10.014

