# Supplementary figures and images for: Robot-assisted total hip arthroplasty following gene therapy for hemophilia a arthropathy: a case report
Source: Arthroplasty. 2025 Dec 3;7:61. doi: 10.1186/s42836-025-00348-5 (PMC12673715; doi:10.1186/s42836-025-00348-5)

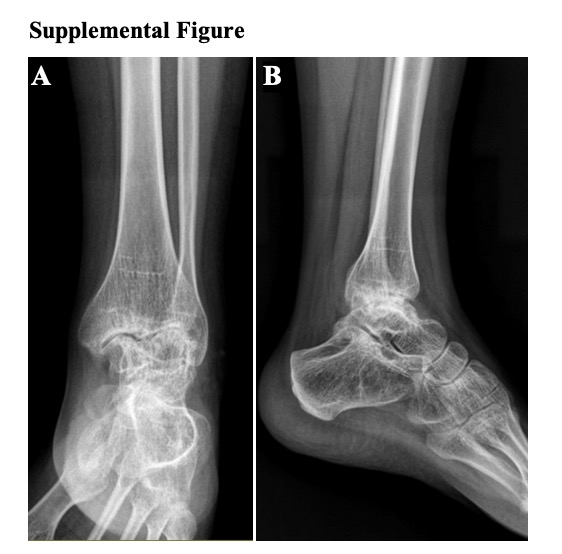

Supplement: Supplementary file 3 — Supplementary Material 3. Fig. S1. Anteroposterior (left) and lateral (right) X-rays of the left ankle joint, revealing hemophilic arthropathy with narrowed joint space. [file 42836_2025_348_MOESM3_ESM.jpg]
